# Independent and Joint Associations of Diet Quality and Rest–Activity Rhythm Phenotypes with Frailty Among Rural Older Adults

**DOI:** 10.3390/nu18213489

**Published:** 2026-10-23

**Authors:** Litao Du, Lina Wang, Ke Chen, Qiang He, Yang Pan, Xianliang Zhang, Si Chen

**Affiliations:** 1 School of Physical Education, Shandong University, Jinan 250061, China; tao7221@outlook.com (L.D.);; 2 Shandong Le Yue Dong Health Management Co., Ltd., Jinan 250101, China; 3 School of Nursing and Rehabilitation, Shandong University, Jinan 250012, China; 4 Department of Physical Education, Tianjin Medical University, Tianjin 300070, China

**Keywords:** frailty, diet quality, circadian rhythm, rest–activity rhythm, older adults

## Abstract

**Objectives:** This study aimed to identify rest–activity rhythm (RAR) phenotypes and examine the independent and joint associations of RAR phenotypes and diet quality with frailty among rural older adults. **Methods**: This cross-sectional study included 1203 rural Chinese adults aged 65 to 85 years. Accelerometer-derived inter-daily stability, intra-daily variability, and relative amplitude were used to identify RAR phenotypes through latent profile analysis. Diet quality was assessed using the China Prime Diet Quality Score, and frailty was assessed using the Fried Frailty Phenotype. RAR phenotypes and median-dichotomized diet quality were combined into joint groups. Robust linear and ordered logistic regression examined associations with frailty score and severity, respectively. Subgroup and interaction analyses were conducted by sex and age group. **Results**: Five RAR phenotypes were identified. Compared with the robust-rhythm phenotype, the severely disrupted phenotype was associated with a 0.516-point higher frailty score and 148.6% higher odds of greater frailty severity, while the fragmented low-amplitude phenotype was associated with 49.3% higher odds. Each 10-point increase in diet quality score was associated with a 0.127-point lower frailty score and 29.3% lower odds of greater frailty severity. Joint analyses showed greater frailty in both typical-rhythm and fragmented low-amplitude phenotypes combined with lower diet quality (β = 0.229, OR = 2.020; β = 0.250, OR = 2.179, respectively), with the highest frailty score observed in the severely disrupted and lower-diet-quality group (β = 0.600; OR = 2.774). Severely disrupted combinations were associated with greater frailty whether diet quality was higher (β = 0.512; OR = 3.092) or lower (β = 0.600; OR = 2.774). No evidence of overall interaction by sex or age group was observed. **Conclusions**: Better diet quality and more robust RAR patterns were independently associated with lower frailty. Considering both factors may provide a broader perspective on behavioral factors related to frailty in older adults.

## 1. Introduction

Frailty is a common geriatric syndrome associated with an increased risk of disability, falls, hospitalization, and death [1]. As a dynamic and potentially reversible condition, frailty can progress to more severe states, making early non-pharmacological interventions particularly important for older adults [2].

Among modifiable lifestyle factors, diet plays an important role in frailty and healthy aging. Lower intakes of protein, vitamin D, and folate, as well as higher intake of added sugars, have been associated with greater frailty among older adults [3,4,5]. By contrast, older adults with adequate intakes of antioxidant-related vitamins, particularly vitamins C and E, tend to have a lower risk of frailty [6]. Greater adherence to healthier dietary patterns, including fruit–vegetable–meat–fish and Mediterranean-style patterns, has also been linked to lower frailty risk [7,8]. Beyond individual nutrients, foods, or dietary patterns, overall diet quality captures the adequacy, balance, and healthfulness of multiple dietary components relative to dietary recommendations. It may therefore provide a more integrated assessment of habitual dietary exposure in relation to frailty. Clinical practice guidelines recommend combining nutrition and physical activity strategies in the management of frailty and pre-frailty among older adults [9].

Circadian rhythms are periodic changes in physiological and behavioral processes regulated by biological clocks [10]. Circadian rhythm disruption has been linked to adverse health outcomes, including metabolic, cardiovascular, neurological, and other age-related disorders [11]. Rest–activity rhythms (RARs), derived from continuous wrist actigraphy, provide an observable behavioral manifestation of 24 h circadian rhythms under free-living conditions. They characterize the stability, fragmentation, and amplitude of daily activity and rest patterns [12]. Emerging evidence has also linked disrupted RARs to frailty. A prospective study found that weaker, less stable, and more fragmented RARs were associated with a higher risk of incident frailty and faster frailty progression [13]. Similarly, a recent NHANES study reported unfavorable associations of lower rhythm strength and greater fragmentation with frailty [14]. However, previous studies have largely examined individual RAR parameters, leaving the co-occurrence of rhythm characteristics within individuals less understood. Recent studies have used clustering approaches to identify distinct RAR patterns associated with cognitive performance, physical fatigability, and mortality [15,16,17], evidence linking such RAR phenotypes to frailty remains limited.

RAR characteristics have been linked to dietary patterns, diet quality, and meal timing [18,19], suggesting that these behaviors may represent related dimensions of daily lifestyle. These factors may also have joint associations with health outcomes. For example, Mediterranean diet adherence and chronotype showed additive associations with depressive symptoms among older adults [20]. Another population-based study suggested that the probability of metabolic syndrome increased from earlier to later chronotypes among individuals with lower adherence to a healthy dietary pattern [21]. However, much of the evidence has relied on self-reported chronotype or sleep timing rather than objective measures of 24 h rest–activity organization. Although actigraphy-derived RARs do not directly measure the endogenous circadian clock, they provide an objective, multi-day assessment of activity and rest patterns under free-living conditions [12]. Whether overall diet quality and objectively identified RAR phenotypes are jointly associated with frailty remains unclear.

Therefore, this study aimed to: (1) identify distinct RAR phenotypes using latent profile analysis; (2) examine the independent associations of RAR phenotypes and overall diet quality with frailty; (3) evaluate their joint associations with frailty and explore whether these associations differed by sex and age group, among rural older adults.

## 2. Materials and Methods

### 2.1. Study Design and Participants

Data for this study was obtained from the baseline survey of the Healthy Aging and Lifestyle Enhancement Project. The study design has been described previously [22]. Briefly, this ongoing cohort study was conducted in rural Shandong Province, China, to investigate health behaviors and their relationships with healthy aging. At baseline, data on health behaviors and related outcomes were collected from 1906 participants. Eligible participants were aged ≥65 years, were able to communicate effectively, and provided written informed consent. Individuals receiving psychotherapy or taking psychotropic medications, such as antidepressants, were excluded. The study was approved by the Ethics Committee of the School of Nursing and Rehabilitation, Shandong University (2024-R-036).

### 2.2. Assessment of 24-Hour Rest–Activity Rhythms

RARs were assessed using a tri-axial accelerometer (ActiGraph wGT3X-BT, Ametris, LLC, Pensacola, FL, USA). Participants were instructed to wear the device on their non-dominant wrist for seven consecutive days and nights, removing it only for showering or other water-based activities. The sampling rate was set at 80 Hz. Raw GT3X files were downloaded using ActiLife v6.13.5 and processed with the R package *GGIR*. Euclidean Norm Minus One was calculated from the raw triaxial acceleration signals and averaged over 5 s epochs, expressed in milligravity units (mg; 1 g = 9.81 m/s^2^). Non-wear time was identified using 60 min windows moving in 15 min increments, with a standard deviation < 3 mg or an acceleration range < 50 mg on at least two axes. Participants were required to have at least three valid days with ≥16 h of wear time per day and a post-calibration error ≤ 0.1 g [23,24]. Three RAR parameters were derived: (1) Inter-daily stability (IS) was calculated as the ratio of the variance in the average daily activity patterns to overall variance. It quantifies day-to-day rhythm stability, with values ranging from 0 to 1. Higher values indicate a stronger alignment with the 24 h circadian activity pattern, reflecting greater rhythm stability. (2) Intra-daily variability (IV) was calculated as the ratio of the mean squared differences between successive hourly activity values to the overall variance. It reflects the within-day fragmentation, with a maximum value of 2. Higher values indicate more frequent transitions between activity and rest. (3) Relative amplitude (RA) was calculated as (M10 − L5)/(M10 + L5), where M10 represents the mean acceleration during the most active 10 h period and L5 represents the mean acceleration during the least active 5 h period. RA ranges from 0 to 1, with higher values indicating a greater contrast between the most active and least active periods of the day. This stronger rhythm may result from increased daytime activity, more restful sleep, or both.

### 2.3. Assessment of Diet Quality

Dietary intake was assessed using a validated semi-quantitative food frequency questionnaire (FFQ), and overall diet quality was evaluated using the China Prime Diet Quality Score (CPDQS) [25].

The FFQ have been described previously [22]. Briefly, it captured habitual intake across 11 major food groups, including staple foods; meat and other animal-source foods; dairy products; legumes; vegetables; fruits; nuts; fried foods; sugar-sweetened beverages; salt; and cooking oil. These groups were further classified into 28 food subcategories comprising more than 80 commonly consumed foods and dietary items. Participants reported their usual consumption frequency and portion size for each item over the previous year. Reported frequencies were converted to daily equivalents and combined with portion size to estimate daily intake (g/day). These values were further winsorized at the 1st and 99th percentiles to reduce the influence of extreme values and potential reporting errors. The food items and classification scheme are detailed in Table S1.

The CPDQS was developed by the Chinese Center for Disease Control and Prevention [25]. Food intakes estimated from the FFQ were mapped to the 22 CPDQS components and scored according to the Chinese Food Guide Pagoda of the Dietary Guidelines for Chinese Residents (2022) [26]. Higher intakes of recommended foods generally received higher scores, whereas foods recommended for limitation were reverse scored. Component scores were summed to obtain a total CPDQS ranging from 0 to 100, with higher scores indicating better overall diet quality and greater adherence to dietary recommendations. Detailed food-group mapping and scoring procedures are provided in Table S2.

### 2.4. Assessment of Frailty

Frailty was assessed using the Fried Frailty Phenotype, which has been adapted and validated in a Chinese population [27]. The phenotype comprises five domains, each contributing one point to a total score from 0 to 5. (1) Exhaustion: Participants were classified as exhausted (1 point) if they reported experiencing either of the following symptoms for at least three days during the past week: (a) “I felt that everything I did was an effort,” or (b) “I could not get going.” (2) Weakness: Grip strength was measured twice in the dominant hand, and the higher value was compared with sex- and BMI-specific cutoffs. Participants were considered weak (1 point) if their grip strength was below or equal to the corresponding cutoff. For men, the cutoffs were ≤29, 30, 32 kg for BMI ≤ 24.0, 24.1–28.0, >28.0 kg/m^2^, respectively. For women, the cutoffs were ≤17.0, 17.3, 18.0, and 21.0 kg for BMI ≤ 23.0, 23.1–26.0, 26.1–29.0, and >29.0 kg/m^2^, respectively. (3) Slowness: Usual walking time over 4.57 m was measured twice, and the shorter time was compared with sex- and height-specific cutoffs. Participants were considered slow (1 point) if their usual walking time met or exceeded the corresponding cutoff. For men, the cutoffs were 7 and 6 s for heights ≤ 173 and >173 cm, respectively. For women, the cutoffs were 7 and 6 s for height ≤ 159 and >159 cm, respectively. (4) Low physical activity: Physical activity energy expenditure was estimated using the International Physical Activity Questionnaire–Short Form. Participants received 1 point for low physical activity if they had <383 kcal/week of activity energy expenditure for men and <270 kcal/week for women. (5) Unintentional weight loss: Participants received 1 point if they reported >4.5 kg or >5% unintentional weight loss relative to body weight during the previous year. Participants were further categorized as robust (0), pre-frail (1–2), or frail (≥3).

### 2.5. Assessment of Covariates

Potential confounders were selected a priori based on the previous literature and established factors associated with dietary quality, RARs, and frailty. Covariates included age, sex, living alone (yes, no), education level (below primary school, primary school, middle school, high school or equivalent or higher), chronic disease (0, 1, ≥2 conditions), smoking status (current, former, never smoker), BMI, and total energy intake. Sleep duration and sleep efficiency were additionally included in the fully adjusted model to account for distinct dimensions of sleep behavior. Chronic disease status was assessed using 29 self-reported chronic conditions, including asthma, coronary heart disease, peptic ulcer, gastroesophageal reflux disease, gastrointestinal disease, liver disease, gallstones, degenerative disk disease, kidney stones, glaucoma, thyroid disease, prostate disease, hypertension, diabetes, stroke, kidney disease, angina, myocardial infarction, cataract, chronic renal failure, Parkinson’s disease, osteoporosis, chronic obstructive pulmonary disease, congestive heart failure, chronic bronchitis, emphysema, arthritis, cancer, and other chronic conditions. Participants were categorized as having no chronic condition, one condition, or two or more chronic conditions (multimorbidity). Sleep duration and sleep efficiency were derived from wrist-worn accelerometer data using the *GGIR* R package. These measures capture related but distinct aspects of sleep behavior and have been associated with frailty in older adults [28,29]. Briefly, the sleep period time window was identified using the HDCZA algorithm, which detects changes in the z-axis angle [30]. Sustained inactivity periods within the window were used to estimate sleep episodes. Sleep duration was calculated as the cumulative duration of these episodes, while sleep efficiency was defined as the percentage of the window classified as sleep.

### 2.6. Statistical Analysis

Participant characteristics were summarized overall and by frailty status. Continuous variables were presented as means and standard deviations and categorical variables as frequencies and percentages. Differences across frailty groups were assessed using one-way analysis of variance for continuous variables and Pearson’s chi-square test for categorical variables. Latent profile analysis (LPA) was performed using standardized IS, IV, and RA z-scores to identify RAR phenotypes. Models with two to eight classes were fitted and evaluated using log-likelihood, Akaike information criterion (AIC), Bayesian information criterion (BIC), Lo–Mendell–Rubin adjusted likelihood ratio test (LMR-LRT), bootstrap likelihood ratio test (BLRT), and entropy. The final solution was selected based on model fit, class size, parsimony, and interpretability. Average posterior probabilities were calculated to assess classification certainty.

Associations of diet quality and RAR phenotypes with frailty were then examined separately. CPDQS was modeled as a continuous variable, with effect estimates reported per 10-point increase. Linear regression with robust standard errors was used to estimate β coefficients and 95% confidence intervals (CIs) for frailty score, while ordered logistic regression estimated odds ratios (ORs) and 95% CIs for frailty severity. The proportional-odds assumption was evaluated using Wald tests of the parallel-lines restrictions. For joint analyses, the five RAR phenotypes were cross-classified with higher and lower diet quality, defined by the median CPDQS, to form 10 groups. The median was chosen as a pragmatic cutoff in the absence of an established clinical threshold and to limit sparse joint categories. The robust-rhythm and higher-diet-quality group served as the common reference. Model 1 adjusted for age, sex, education, living arrangement, BMI, smoking status, and comorbidity. Model 2 additionally adjusted for total energy intake, and Model 3 further adjusted for sleep duration and efficiency.

Several sensitivity analyses were performed. When evidence of non-proportionality was detected, partial proportional-odds (PPO) models with robust standard errors were fitted to assess the implications of relaxing these restrictions. In addition, the Benjamini–Hochberg false discovery rate (FDR) procedure was applied to account for multiple comparisons across RAR phenotypes [31]. The Bolck–Croon–Hagenaars (BCH) approach was used to account for LPA classification uncertainty [32]. Posterior class-membership probabilities were used to derive the classification-error matrix and BCH weights, which were applied in fully adjusted models. In addition, to assess potential overlap between RAR phenotypes and the low-physical-activity criterion of frailty, we recalculated the frailty score excluding this criterion (range, 0–4) and repeated the fully adjusted joint analysis.

Subgroup analyses were conducted by sex and age group (65–74 and ≥75 years) using fully adjusted linear and ordered logistic regression models with robust standard errors. Overall interaction significance was evaluated using Wald tests. All analyses were conducted using R (version 4.3.1) and Stata (version 16.0; StataCorp, College Station, TX, USA). Statistical significance was defined as a two-sided *p* value < 0.05.

## 3. Results

### 3.1. Baseline Characteristic of the Participants

Of the 1906 baseline participants, 1203 were included in the final analysis after exclusions detailed in Figure S1. Among them, 42.5% were pre-frail, and 2.8% were frail. Participants with greater frailty severity tended to be older, were more likely to be male, had more chronic conditions, and had lower diet quality (all *p* < 0.001). Greater frailty severity was also associated with higher IV and lower IS and RA (all *p* < 0.001). Smoking status differed significantly across frailty groups (*p* < 0.001), whereas no significant differences were observed for other characteristics (Table 1).

### 3.2. Results of Latent Profile Analysis

**Figure 1:**
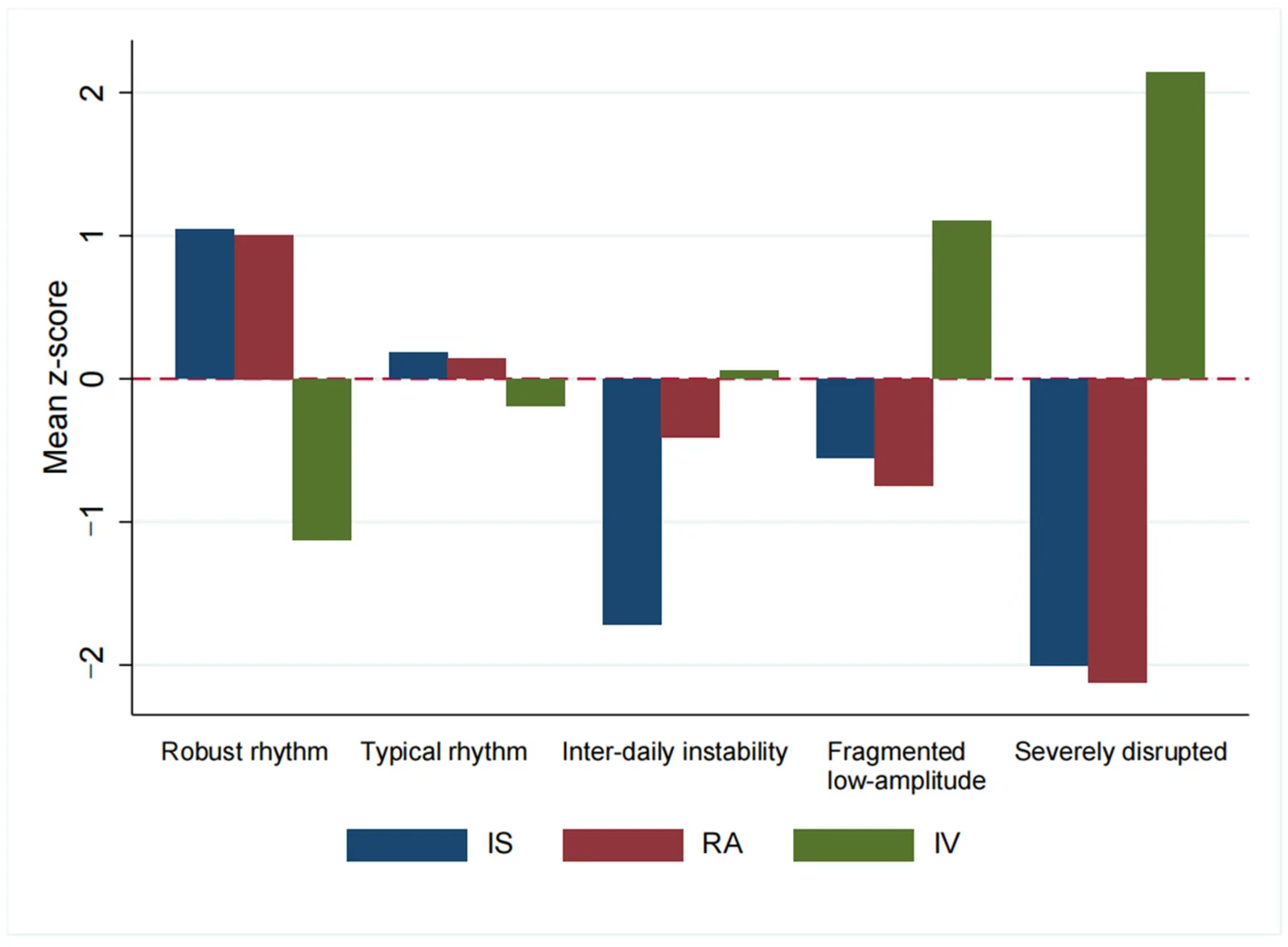
Characteristics of five rest–activity rhythm phenotypes.

Across the two- to eight-class solutions, log-likelihood increased and AIC decreased with additional classes, whereas BIC was lowest for the five-class solution (9204.33; Table S3). The smallest class in the five-class solution included 61 participants (5.1%), while the four- and six- to eight-class solutions contained classes representing less than 5% of the sample. The five-class solution was retained based on model fit, classification certainty, class size, parsimony, and interpretability, with average posterior probabilities ranging from 0.704 to 0.913 (Table S3). The five RAR phenotypes were characterized as follows (Table 2 and Figure 1). (1) Inter-daily instability phenotype (5.4%): Markedly lower IS, with relatively modest differences in IV and RA, indicating poor day-to-day rhythm regularity. (2) Fragmented low-amplitude phenotype (18.8%): Higher IV, lower RA, and moderately reduced IS, reflecting greater fragmentation and weaker differentiation between active and rest periods. (3) Typical-rhythm phenotype (51.0%): IS, IV, and RA values close to the overall sample averages, representing an intermediate rhythm profile without marked disruption or robustness. (4) Severely disrupted phenotype (5.1%): The lowest IS and RA and highest IV, indicating pronounced day-to-day irregularity, fragmentation, and reduced rhythm amplitude. (5) Robust-rhythm phenotype (19.7%): The highest IS and RA and lowest IV, reflecting regular, less fragmented rest–activity patterns with a clear contrast between active and rest periods.

### 3.3. Association of Rest–Activity Rhythm Phenotypes with Frailty

Compared with the robust-rhythm phenotype, the fragmented low-amplitude phenotype was associated with a 0.135-point higher frailty score (95% CI: −0.001 to 0.271) and 49.3% higher odds of greater frailty severity (OR = 1.493, 95% CI: 1.017–2.191) in the fully adjusted model. Stronger associations were observed for the severely disrupted phenotype, with a 0.516-point higher frailty score (95% CI: 0.205–0.826) and 148.6% higher odds of greater frailty severity (OR = 2.486, 95% CI: 1.185–5.218). Neither the inter-daily instability nor the typical-rhythm phenotype was significantly associated with frailty (Table 3).

### 3.4. Association of Dietary Quality with Frailty

Higher diet quality was consistently associated with lower frailty across all models (Table 4). In the fully adjusted model, each 10-point increase in CPDQS was associated with a 0.127-point lower frailty score (95% CI: −0.180 to −0.073) and 29.3% lower odds of greater frailty severity (OR = 0.707, 95% CI: 0.610–0.818).

### 3.5. Joint Association of Diet Quality, Rest–Activity and Rhythm Phenotypes with Frailty

**Figure 2:**
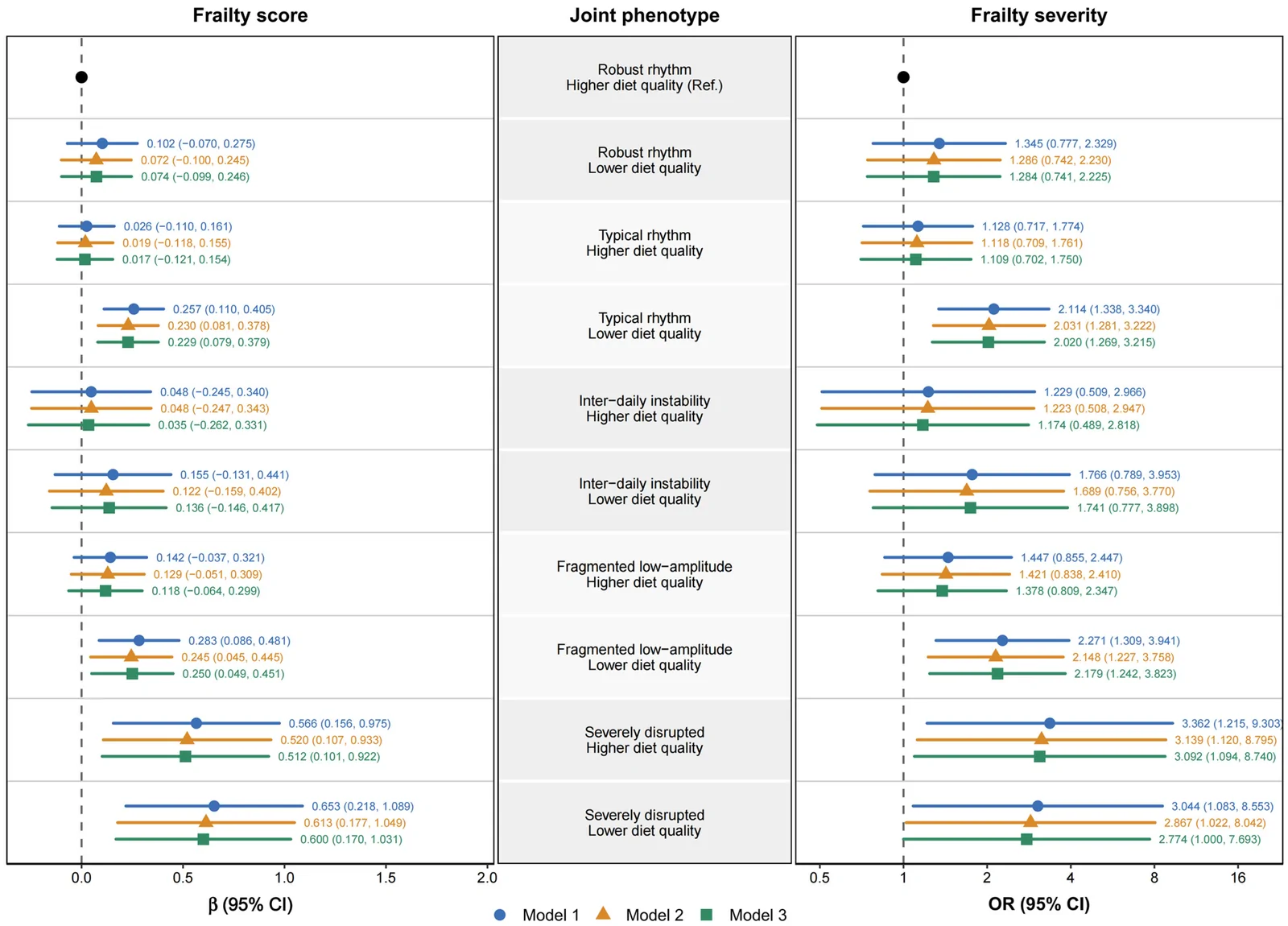
Joint associations of diet quality and rest–activity rhythm phenotypes with frailty score and frailty severity.

As shown in Figure 2, associations were generally consistent across adjustment models. Compared with the robust-rhythm and higher-diet-quality group, the fragmented low-amplitude and lower-diet-quality group had a higher frailty score (β = 0.250, 95% CI: 0.049–0.451) and greater odds of more severe frailty (OR = 2.179, 95% CI: 1.242–3.823) in the fully adjusted model. Similar associations were observed for the typical-rhythm and lower-diet-quality group (β = 0.229, 95% CI: 0.079–0.379; OR = 2.020, 95% CI: 1.269–3.215). The largest estimates were observed in the severely disrupted rhythm groups. The severely disrupted and higher-diet-quality group had a 0.512-point higher frailty score (95% CI: 0.101–0.922) and greater odds of more severe frailty (OR = 3.092, 95% CI: 1.094–8.740). Corresponding estimates for the severely disrupted and lower-diet-quality group were β = 0.600 (95% CI: 0.170–1.031) and OR = 2.774 (95% CI: 1.000–7.693). Estimates for the remaining joint groups were generally smaller.

### 3.6. Sensitivity Analysis

Proportional-odds diagnostics identified departures from the assumption for selected predictors in all three ordered logistic models. After relaxing the corresponding constraints, the remaining parallel-lines constraints were not rejected in all models. PPO estimates for predictors retaining the proportional-odds constraints were generally similar to those from the original models, whereas some threshold-specific estimates were extreme and potentially unstable (Table S5). For FDR correction, the associations of higher diet quality and the severely disrupted RAR phenotype with frailty remained statistically significant (Table S6). However, the association between the fragmented low-amplitude phenotype and frailty severity and those of the severely disrupted and higher-diet-quality group with frailty score and severity were no longer statistically significant (FDR-adjusted *p* > 0.05). BCH analyses accounting for classification uncertainty yielded estimates similar in direction and magnitude to the primary analyses, although some confidence intervals were wider (Table S7). Excluding the low-physical-activity criterion from the frailty score also produced broadly consistent results (Table S8).

### 3.7. Subgroup Analysis

Subgroup analyses (Tables S9 and S10) showed numerically larger estimates among women than men, but several estimates had wide confidence intervals. There was no evidence of overall interaction by sex (*p* for interaction = 0.508 for frailty score and 0.448 for frailty severity) or age group (*p* for interaction = 0.548 and 0.444, respectively).

## 4. Discussion

This study identified five distinct RAR phenotypes and found that poorer diet quality and disrupted RAR patterns were independently associated with greater frailty among rural older adults. In joint analyses, the severely disrupted phenotype was associated with greater frailty regardless of diet quality, whereas the fragmented low-amplitude and typical-rhythm phenotypes were also associated with greater frailty when combined with lower diet quality. These findings provide a more comprehensive perspective on the joint relevance of diet quality and 24 h rest–activity organization to frailty in older adults.

The five RAR phenotypes captured distinct patterns of 24 h rest–activity organization, ranging from robust rhythms characterized by high inter-daily stability and day–night amplitude and low intra-daily variability to severely disrupted phenotypes showing the opposite pattern. Study of older NHANES participants identified four profiles; the unstable, low-amplitude, and delayed profile was associated with higher mortality [15]. Another clustering study indicated that higher intra-daily variability, lower inter-daily stability, and desynchronized acrophase pattern were associated with severe depressive symptoms and a higher BMI [33]. While previous studies of frailty have mainly examined individual RAR dimensions [13,14,34], our findings provide further insight into how different patterns of RAR relate to frailty. The robust-rhythm phenotype, characterized by greater day-to-day regularity, lower within-day fragmentation, and stronger activity–rest contrast, showed the most favorable frailty profile, whereas the severely disrupted phenotype showed the strongest association with greater frailty. The fragmented low-amplitude phenotype was also associated with greater frailty, suggesting that greater within-day fragmentation and reduced activity–rest contrast may be relevant to frailty even in the absence of marked day-to-day instability. Notably, the inter-daily instability phenotype, mainly characterized by reduced inter-daily stability, was not associated with frailty; this suggests that impaired day-to-day regularity alone may be less strongly related to frailty. In summary, the present findings emphasize the coexistence of disturbances across multiple RAR dimensions and may provide additional information beyond abnormalities in a single rhythm characteristic.

Higher overall diet quality was consistently associated with lower frailty, in line with previous evidence. A recent systematic review and dose–response meta-analysis reported an inverse association between diet quality, assessed using the Healthy Eating Index, and frailty risk [35]. Longitudinal studies in Chinese adults have also linked adherence to healthier dietary indices, including the Chinese Healthy Eating Index, AHEI, and DASH, to slower frailty progression [36]. A recent study among Chinese rural older adults also reported that greater adherence to a milk–bean–egg–fruit dietary pattern was associated with a lower risk of frailty [37]. Our findings add to this evidence by evaluating diet quality using the CPDQS, which reflects adherence to Chinese dietary recommendations and provides a culturally relevant assessment of overall dietary quality.

The joint analysis provided further insight into the associations of diet quality and RAR phenotypes with frailty. Both categories combining the severely disrupted phenotype with higher or lower diet quality were associated with greater frailty relative to the robust-rhythm and higher-diet-quality reference group. This finding highlights the relevance of disturbances in daily rest–activity organization to frailty, even among individuals with relatively higher diet quality. The typical-rhythm and fragmented low-amplitude phenotypes combined with lower diet quality were also associated with greater frailty. This suggests that a rhythm phenotype without marked disruption does not necessarily correspond to a favorable frailty profile. In particular, diet quality and RAR phenotypes capture complementary aspects of daily lifestyle that may not be adequately characterized by either factor alone. Considering their combinations may therefore provide a more comprehensive description of behavioral profiles associated with frailty and help identify patterns that warrant further investigation. Notably, we conducted interaction tests between RAR phenotypes and diet quality with no evidence of interaction for either frailty score (*p* for interaction = 0.778) or frailty severity (*p* for interaction = 0.752). Thus, the observed joint associations should not be interpreted as evidence that diet quality modifies the associations between RAR phenotypes and frailty. In addition, neither category involving the inter-daily instability phenotype showed a significant association, consistent with the RAR-only analysis. This suggests that isolated impairment in day-to-day regularity, in the absence of marked fragmentation or amplitude reduction, may be less strongly associated with frailty and may consequently also be less sensitive to the differences in diet quality. However, this should be interpreted cautiously due to the relatively small number of participants in this phenotype.

Subgroup analyses showed numerical differences across sex and age groups, but formal interaction tests provided no evidence of effect modification. Previous research has reported stronger associations between lower relative amplitude and frailty among women [14], while age-related changes in RAR organization have also been documented [38]. However, several estimates in our subgroup analysis had extremely wide confidence intervals, indicating considerable uncertainty in the magnitude of these associations, suggesting limited precision despite the large point estimate. Given the small number of frail participants, further stratification by sex and age across multiple joint categories may have resulted in sparse data and potentially unstable estimates. Therefore, the apparent differences across subgroups should be regarded as exploratory and require confirmation in larger studies.

Several limitations should be considered. The cross-sectional design precludes causal inference and cannot exclude reverse causation. FFQ-based dietary assessment is subject to recall and measurement error, while local adaptations to CPDQS scoring may limit comparability with studies using the original score. In addition, recruitment from rural areas of a single province and differences in sex distribution and smoking status between included and excluded participants (Table S11) may introduce selection bias and limit the generalizability of our findings. The small number of frail participants (*n* = 34), particularly within joint exposure categories, limited the precision of ordinal regression estimates and subgroup analysis. Although proportional-odds diagnostics identified departures for two joint categories, a more flexible partial proportional-odds model produced unstable estimates. The corresponding findings should therefore be interpreted cautiously. However, the similar pattern observed for analysis of frailty score provides additional support. This study also has several strengths. Multi-day wrist-worn accelerometry provided objective RAR assessment, while GGIR enabled standardized data processing, thereby facilitating cross-study comparability. Several sensitivity analyses provided complementary assessments of the robustness of the findings.

## 5. Conclusions

Higher diet quality and more robust RAR patterns were associated with lower frailty in rural older adults. The combinations of the severely disrupted phenotype with either higher or lower diet quality, and lower diet quality with either the fragmented low-amplitude or typical-rhythm phenotype, were all associated with greater frailty. These findings provide a new perspective on the associations of diet quality and 24 h rest–activity organization with frailty in older adults.

## Figures and Tables

**Table 1:** Baseline characteristics of the participants.

Characteristics	Total (*N* = 1203)	Robust (*N* = 658)	Pre-Frail (*N* = 511)	Frail (*N* = 34)	*p*-Value
Age, years	72.49 (4.17)	71.89 (4.09)	73.14 (4.17)	74.53 (3.65)	<0.001
Sex, *N* (%)					<0.001
Male	525 (43.64)	246 (37.39)	258 (50.49)	21 (61.76)	
Female	678 (56.36)	412 (62.61)	253 (49.51)	13 (38.24)	
BMI, kg/m^2^	24.56 (3.58)	24.47 (3.28)	24.62 (3.92)	25.53 (4.01)	0.221
Education, *N* (%)					0.236
Below primary school	728 (60.52)	389 (59.12)	318 (62.23)	21 (61.76)	
Primary school	231 (19.20)	123 (18.69)	101 (19.77)	7 (20.59)	
Middle school	190 (15.79)	119 (18.09)	68 (13.31)	3 (8.82)	
High school, equivalent or higher	54 (4.49)	27 (4.10)	24 (4.70)	3 (8.82)	
Living alone, *N* (%)					0.188
No	970 (80.63)	541 (82.22)	400 (78.28)	29 (85.29)	
Yes	233 (19.37)	117 (17.78)	111 (21.72)	5 (14.71)	
Smoking status, *N* (%)					<0.001
Current smoker	211 (17.54)	106 (16.11)	100 (19.57)	5 (14.71)	
Former smoker	236 (19.62)	108 (16.41)	114 (22.31)	14 (41.18)	
Non-smoker	756 (62.84)	444 (67.48)	297 (58.12)	15 (44.12)	
Chronic disease status, *N* (%)					<0.001
No chronic condition	283 (23.52)	171 (25.99)	111 (21.72)	1 (2.94)	
One chronic condition	379 (31.50)	230 (34.95)	142 (27.79)	7 (20.59)	
≥2 chronic conditions	541 (44.98)	257 (39.06)	258 (50.49)	26 (76.47)	
Total energy intake, kcal/day	1947.39 (765.24)	1950.00 (757.91)	1959.44 (778.13)	1715.78 (691.73)	0.197
CPDQS	63.08 (8.85)	64.25 (8.69)	61.94 (8.78)	57.40 (8.87)	<0.001
IV	0.784 (0.208)	0.765 (0.199)	0.800 (0.212)	0.907 (0.248)	<0.001
IS	0.674 (0.115)	0.684 (0.109)	0.668 (0.116)	0.582 (0.136)	<0.001
RA	0.736 (0.089)	0.747 (0.084)	0.727 (0.091)	0.659 (0.093)	<0.001
Sleep duration, min/day	326.69 (67.54)	324.11 (65.15)	329.38 (69.85)	335.96 (76.91)	0.300
Sleep efficiency, %	78.66 (9.21)	78.82 (8.99)	78.40 (9.54)	79.59 (8.20)	0.614
Frailty score	0.61 (0.79)	0 (0)	1.22 (0.42)	3.15 (0.44)	<0.001

Note: Data are presented as mean (SD) for continuous variables and *N* (%) for categorical variables. BMI, body mass index; CPDQS, China Prime Diet Quality Score; IV, intra-daily variability; IS, inter-daily stability; RA, relative amplitude.

**Table 2:** Characteristics of the five rest–activity rhythm phenotypes identified by latent profile analysis.

Rest–Activity Rhythm Phenotype	IS	IV	RA	Participants, *N* (%)
Inter-daily instability	0.478 (0.056)	0.796 (0.103)	0.699 (0.071)	65 (5.4)
Fragmented low amplitude	0.611 (0.070)	1.013 (0.116)	0.670 (0.061)	226 (18.8)
Typical rhythm	0.695 (0.069)	0.744 (0.102)	0.748 (0.060)	614 (51.0)
Severely disrupted	0.445 (0.083)	1.228 (0.165)	0.547 (0.070)	61 (5.1)
Robust rhythm	0.794 (0.059)	0.551 (0.098)	0.825 (0.049)	237 (19.7)

Note: Values precented as mean (SD) unless otherwise indicated. IS, inter-daily stability; IV, intra-daily variability; RA, relative amplitude.

**Table 3:** Associations of rest–activity rhythm phenotypes with frailty in rural older adults.

Outcome	Phenotype	Model 1		Model 2		Model 3	
Frailty score		β (95% CI)	*p*-value	β (95% CI)	*p*-value	β (95% CI)	*p*-value
	Robust rhythm	Reference					
	Inter-daily instability	0.048 (−0.160, 0.256)	0.648	0.043 (−0.162, 0.248)	0.683	0.045 (−0.161, 0.250)	0.671
	Fragmented low amplitude	0.153 (0.017, 0.288)	0.028	0.139 (0.003, 0.274)	0.045	0.135 (−0.001, 0.271)	0.051
	Typical rhythm	0.081 (−0.023, 0.185)	0.125	0.078 (−0.025, 0.181)	0.140	0.076 (−0.027, 0.179)	0.150
	Severely disrupted	0.561 (0.247, 0.876)	<0.001	0.526 (0.211, 0.841)	0.001	0.516 (0.205, 0.826)	0.001
		OR (95% CI)	*p*-value	OR (95% CI)	*p*-value	OR (95% CI)	*p*-value
Frailty severity	Robust rhythm	Reference					
	Inter-daily instability	1.269 (0.701, 2.296)	0.431	1.259 (0.699, 2.269)	0.443	1.261 (0.700, 2.272)	0.439
	Fragmented low amplitude	1.536 (1.049, 2.247)	0.027	1.500 (1.023, 2.200)	0.038	1.493 (1.017, 2.191)	0.041
	Typical rhythm	1.310 (0.953, 1.802)	0.097	1.305 (0.949, 1.793)	0.101	1.298 (0.944, 1.783)	0.108
	Severely disrupted	2.709 (1.284, 5.718)	0.009	2.540 (1.203, 5.363)	0.014	2.486 (1.185, 5.218)	0.016

Note: Model 1 was adjusted for age, sex, education, living alone, BMI, smoking status, and chronic disease status. Model 2 was additionally adjusted for total energy intake. Model 3 was further adjusted for sleep duration and sleep efficiency. CI, confidence interval; OR, odds ratio; BMI, body mass index.

**Table 4:** Associations of dietary quality with frailty in rural older adults.

Outcome	Model 1		Model 2		Model 3	
	β (95% CI)	*p*-value	β (95% CI)	*p*-value	β (95% CI)	*p*-value
Frailty score	−0.140 (−0.192, −0.089)	<0.001	−0.125 (−0.178, −0.072)	<0.001	−0.127 (−0.180, −0.073)	<0.001
	OR (95% CI)	*p*-value	OR (95% CI)	*p*-value	OR (95% CI)	*p*-value
Frailty severity	0.695 (0.604, 0.801)	<0.001	0.709 (0.613, 0.821)	<0.001	0.707 (0.610, 0.818)	<0.001

Note: Model 1 was adjusted for age, sex, education, living arrangement, body mass index, smoking status, and comorbidity. Model 2 was additionally adjusted for total energy intake. Model 3 was further adjusted for sleep duration and sleep efficiency. CPDQS was modeled per 10-point increase. CI, confidence interval; OR, odds ratio.

## Data Availability

The data that support the findings of this study are available from the corresponding author upon reasonable request.

## Supplementary Materials

The following supporting information can be downloaded at https://www.mdpi.com/article/10.3390/nu18213489/s1, Table S1: Food groups and items used for CPDQS calculation based on the food frequency questionnaire; Table S2: CPDQS scoring components and cut-off values; Table S3: Fitting indices of 2 to 8 class; Table S4: Average posterior probability matrix for the selected five-class solution; Table S5: Comparison of proportional-odds and partial proportional-odds model estimates for the associations of rest–activity rhythm phenotypes and diet quality with frailty severity; Table S6: Raw and FDR-adjusted *p* value of the main analysis; Table S7: BCH-weighted regression sensitivity analyses accounting for latent-profile classification uncertainty; Table S8: Sensitivity analysis of the joint associations between diet quality and RAR phenotypes with a modified Fried frailty score excluding the low-physical-activity component; Table S9: Sex-stratified associations of joint diet quality and rest–activity rhythm phenotypes with frailty; Table S10: Age-stratified associations of joint diet quality and rest–activity rhythm phenotypes with frailty; Figure S1: Participant flow diagram; Table S11: Differences between included and excluded participants due to missing data.

## Abbreviations

The following abbreviations are used in this manuscript:

RAR	Rest–activity rhythm
IV	Intra-daily variability
IS	Inter-daily stability
RA	Relative amplitude
FFQ	Food frequency questionnaire
CPDQS	China Prime Diet Quality Score
LPA	Latent profile analysis
