# Supplementary material for: Independent and Joint Associations of Diet Quality and Rest–Activity Rhythm Phenotypes with Frailty Among Rural Older Adults
Source: Nutrients. 2026 Oct 23;18(21):3489. doi: 10.3390/nu18213489 (PMC13764868; doi:10.3390/nu18213489)
Supplement: Supplementary file 1 [file nutrients-18-03489-s001.zip › nutrients-4577235-supplementary.pdf]

Table S1. Food groups and items used for CPDQS calculation based on the food frequency questionnaire.

Category	Sub-category	Detailed description
1. Staple Foods	a. Rice	White rice, rice porridge, fried rice, etc.
	b. Wheat Products	Steamed buns (Mantou), noodles, stuffed buns (Baozi), flatbreads, etc.
	c. Coarse Grains	Millet, corn, sorghum, oats, etc.
	d. Sweet Potato	Sweet potato
	e. Other Tubers	Potato, Chinese yam, taro, etc.
2. Animal-Based Foods	a. Livestock Meat and Products	Pork, beef, mutton, and their processed products
	b. Poultry and Products	Chicken, duck, goose, and their processed products
	c. Aquatic/Seafood Products	Fish, shrimp, crab, shellfish, etc. (fresh or frozen)
	d. Eggs	Chicken eggs, duck eggs, etc.
3. Dairy Products	a. Whole Milk/Yogurt	—
	b. Whole Milk Powder	—
	c. Skim Milk/Yogurt	—
	d. Skim Milk Powder	—
4. Legumes	a. Dry Beans	Soybeans, adzuki beans, mung beans, green beans, black beans, etc.
	b. Bean Products	Firm tofu, soft tofu, tofu skin, dried tofu sticks, tofu pudding, etc., excluding soy milk and other soy-based beverages
	c. Soy Milk	—
5. Vegetables	a. Total Vegetables	Leafy greens (e.g., bok choy), spinach, water spinach, tomatoes, green peppers, carrots, Chinese cabbage, radish, cabbage, etc., excluding potatoes, Chinese yam, and other tubers
	b. Dark Green Vegetables	Spinach, Chinese flowering cabbage (youcai), garland chrysanthemum, lettuce, etc.
	c. Red/Yellow/Orange Vegetables	Tomatoes, bell peppers, carrots, red-hearted radish, etc.
6. Fruits	a. Total Fruits	Apples, bananas, oranges, pears, etc.
	b. Dark Fruits	Brightly colored fruits (e.g., red, orange, yellow, blue, and purple), such as oranges/mandarins, mangoes, pineapples, red-fleshed dragon fruit, mulberries, blueberries, strawberries, and cherries
	c. Citrus Fruits	Oranges, pomelos, tangerines, grapefruits, etc.
7. Nuts	—	Melon seeds, peanuts, walnuts, etc.
8. Fried Foods	—	Deep-fried dough sticks (Youtiao), fried pancakes, French fries, potato chips, fried chicken wings, fried fish, fried dough twists (Mahua), etc.
9. Sugar-Sweetened Beverages	—	Cola, Sprite, iced black tea, etc.
10. Salt	—	Cooking salt
11. Cooking Oil	a. Vegetable Oil	Peanut oil, corn oil, etc.
	b. Animal Fat	Lard, tallow, etc.

Table S2. CPDQS scoring components and cut-off values.

Positively Scored Food Components (0–4 Points)					
Food Group	0 Points	1 Point	2 Points	3 Points	4 Points
Red/Yellow/Orange Vegetables	0	(0, 20)	[20, 40)	[40, 60)	≥60
Other vegetables	0	(0, 40)	[40, 80)	[80, 120)	≥120
Dark fruits	0	(0, 20)	[20, 40)	[40, 60)	≥60
Citrus fruits	0	(0, 20)	[20, 40)	[40, 60)	≥60
Other fruits	0	(0, 20)	[20, 40)	[40, 60)	≥60
Nuts	0	(0, 4)	[4, 8)	[8, 12)	≥12
Poultry and Products	0	(0, 10)	[10, 20)	[20, 30)	≥30
Dairy Products	0	(0, 60)	[60, 120)	[120, 180)	≥180
Eggs	0	(0, 10)	[10, 20)	[20, 30)	≥30
Positively Scored Food Components (0–8 Points)					
Food Group	0 Points	2 Points	4 Points	6 Points	8 Points
Dark green vegetables	0	(0, 20)	[20, 40)	[40, 60)	≥60
Bean Products/Soy Milk	0	(0, 4)	[4, 8)	[8, 12)	≥12
Aquatic/Seafood Products	0	(0, 10)	[10, 20)	[20, 30)	≥30
Coarse Grains/Dry Beans	0	(0, 10)	[10, 20)	[20, 30)	≥30
Tubers (0–2 Points)					
Food Group	0 Points	0.5 Points	1 Point	1.5 Points	2 Points
Sweet potatoes	0	(0, 20)	[20, 40)	[40, 60)	≥60
Other tubers	0	(0, 20)	[20, 40)	[40, 60)	≥60
Reverse-Scored Food Components					
Food Group	4 Points	3 Points	2 Points	1 Point	0 Points
Livestock Meat and Products	[0, 50]	(50, 100]	(100, 150]	(150, 200]	>200
Fried foods	[0, 50]	(50, 100]	(100, 150]	(150, 200]	>200
Rice/Wheat Products	(0, 150]	(150, 300]	(300, 450]	(450, 600]	>600 or 0
Sugar-sweetened beverages	[0, 100]	(100, 200]	(200, 300]	(300, 400]	>400
Cooking oil	[0, 25]	(25, 50]	(50, 75]	(75, 100]	>100
Salt	[0, 5]	(5, 10]	(10, 15]	(15, 20]	>20
Alcohol	[0, 15]	(15, 30]	(30, 45]	(45, 60]	>60

Note: All intake values are expressed in grams per day (g/day) based on edible portions. Scores were assigned according to the corresponding intake range for each food group. Soybeans and related products were calculated based on dry soybean equivalent, and soy products were converted according to protein content. Dairy intake was expressed as liquid milk equivalent, and other dairy products were converted according to protein content. Alcohol intake was calculated as ethanol equivalent based on beverage-specific alcohol concentration.

Table S3. Fitting indices of 2 to 8 class.

Classes	LL	AIC	BIC	Entropy	LMR-LRT *P-value*	BLRT *P-value*	Smallest class, n (%)
2	-4719.003	9458.005	9508.93	0.764	—	0.001	298 (24.77)
3	-4571.424	9170.849	9242.15	0.752	<0.001	0.001	79 (6.57)
4	-4543.457	9122.915	9214.58	0.663	<0.001	0.001	60 (4.99)
5	***-4524.147***	***9092.294***	***9204.33***	***0.693***	***<0.001***	***0.001***	***61 (5.07)***
6	-4516.137	9084.275	9216.68	0.649	0.004	0.011	47 (3.91)
7	-4503.043	9066.086	9218.86	0.673	<0.001	0.196	19 (1.58)
8	-4487.514	9043.028	9216.18	0.688	<0.001	0.006	13 (1.08)

Note: LL, log-likelihood; AIC, Akaike information criterion; BIC, Bayesian information criterion; LMR-LRT, Lo–Mendell–Rubin adjusted likelihood ratio test; BLRT, bootstrap likelihood ratio test. Bold values indicate the selected solution.

Table S4. Average posterior probability matrix for the selected five-class solution.

Modal-assigned RAR phenotype	Typical rhythm	Inter-daily instability	Fragmented low-amplitude	Robust rhythm	Severely disrupted
Typical rhythm	**0.805**	0.030	0.054	0.111	0.000
Inter-daily instability	0.128	**0.704**	0.159	0.000	0.008
Fragmented low-amplitude	0.126	0.091	**0.759**	0.000	0.023
Robust rhythm	0.191	0.000	0.000	**0.809**	0.000
Severely disrupted	0.000	0.010	0.077	0.000	**0.913**

Note: RAR, rest–activity rhythm. The diagonal values represent the average maximum posterior probability of each latent phenotype. Non-diagonal values represent the probability of misclassification. Bold diagonal values represent the class-specific average posterior probabilities. Values may not sum exactly to 1.000 because of rounding.

Table S5. Comparison of proportional-odds and partial proportional-odds model estimates for the associations of rest–activity rhythm phenotypes and diet quality with frailty severity.

Exposure		Ordered logistic OR (95% CI)	PPO Threshold 1 (pre-frail/frail vs. robust) OR (95% CI)	PPO Threshold 2 (frail vs. robust/pre-frail) OR (95% CI)
RAR phenotypes	Robust rhythm	Reference	Reference	Reference
	Inter-daily instability	1.261 (0.700–2.272)	1.270 (0.700–2.304)	—
	Fragmented low-amplitude	1.493 (1.017–2.191)	1.511 (1.025–2.227)	—
	Typical rhythm	1.298 (0.944–1.783)	1.306 (0.948–1.799)	—
	Severely disrupted	2.486 (1.185–5.218)	1.750 (0.939–3.263)	8.284 (3.493–19.648)
Diet quality	CPDQS (per 10-point increase)	0.707 (0.610–0.818)	0.728 (0.630–0.842)	0.483 (0.325–0.718)
Joint categories	Robust rhythm + Higher diet quality	Reference	Reference	Reference
	Robust rhythm + Lower diet quality	1.284 (0.741–2.225)	1.376 (0.782–2.420)	2.43×10^{-6} (1.33×10^{-6}–4.43×10^{-6})*
	Typical rhythm + Higher diet quality	1.109 (0.702–1.750)	1.110 (0.699–1.761)	—
	Typical rhythm + Lower diet quality	2.020 (1.269–3.215)	2.020 (1.263–3.231)	—
	Inter-daily instability + Higher diet quality	1.174 (0.489–2.818)	1.123 (0.461–2.734)	—
	Inter-daily instability + Lower diet quality	1.741 (0.777–3.898)	1.776 (0.791–3.985)	—
	Fragmented low-amplitude + Higher diet quality	1.378 (0.809–2.347)	1.426 (0.829–2.453)	—
	Fragmented low-amplitude + Lower diet quality	2.179 (1.242–3.823)	2.165 (1.230–3.812)	—
	Severely disrupted + Higher diet quality	3.092 (1.094–8.740)	3.334 (1.116–9.962)	—
	Severely disrupted + Lower diet quality	2.774 (1.000–7.693)	1.646 (0.775–3.497)	12.724 (4.880–33.177)

Notes: *The Threshold 2 estimate for the Robust rhythm + Lower diet quality group was unstable (extremely small OR). Abbreviations: CI, confidence interval; CPDQS, China Prime Diet Quality Score; OR, odds ratio; PPO, partial proportional-odds; RAR, rest–activity rhythm.

Table S6. Raw and FDR -adjusted *P* value of the main analysis.

Frailty measure	Independent Variables	*P* value	FDR-adjusted *P* value
Frailty score	Robust rhythm	Reference	
	Inter-daily instability	0.671	0.671
	Fragmented low-amplitude	0.051	0.085
	Typical rhythm	0.150	0.188
	Severely disrupted	0.001	0.004
Frailty severity	Robust rhythm	Reference	
	Inter-daily instability	0.440	0.488
	Fragmented low-amplitude	0.041	0.082
	Typical rhythm	0.108	0.154
	Severely disrupted	0.016	0.040
Frailty score	CPDQS per 10-point increase	<0.001	<0.001
Frailty severity		<0.001	<0.001
Frailty score	Robust rhythm + Higher diet quality	Reference	
	Robust rhythm + Lower diet quality	0.349	0.448
	Inter-daily instability + Higher diet quality	0.921	0.921
	Inter-daily instability + Lower diet quality	0.331	0.448
	Fragmented low-amplitude + Higher diet quality	0.184	0.331
	Fragmented low-amplitude + Lower diet quality	0.018	0.055
	Typical rhythm + Higher diet quality	0.839	0.889
	Typical rhythm + Lower diet quality	0.004	0.034
	Severely disrupted + Higher diet quality	0.015	0.052
	Severely disrupted + Lower diet quality	0.008	0.036
Frailty severity	Robust rhythm + Higher diet quality	Reference	
	Robust rhythm + Lower diet quality	0.319	0.448
	Inter-daily instability + Higher diet quality	0.807	0.889
	Inter-daily instability + Lower diet quality	0.167	0.331
	Fragmented low-amplitude + Higher diet quality	0.209	0.342
	Fragmented low-amplitude + Lower diet quality	0.008	0.036
	Typical rhythm + Higher diet quality	0.670	0.804
	Typical rhythm + Lower diet quality	0.004	0.034
	Severely disrupted + Higher diet quality	0.031	0.081
	Severely disrupted + Lower diet quality	0.054	0.121

Note: FDR, the Benjamini–Hochberg false discovery rate. CPDQS, Chinese Healthy Eating Index–based Prospective Dietary Quality Score; FDR, false discovery rate; RAR, rest–activity rhythm. Effect estimates and 95% confidence intervals from the models were presented in Figure 2.

Table S7. BCH-weighted regression sensitivity analyses accounting for latent-profile classification uncertainty.

Exposures	BCH-weighted β (95% CI)	*P* value	BCH-weighted OR (95% CI)	*P* value
RAR phenotype				
Robust rhythm	Reference	—	Reference	—
Inter-daily instability	0.032 (−0.266, 0.330)	0.833	1.282 (0.540, 3.044)	0.573
Fragmented low-amplitude	0.176 (−0.013, 0.364)	0.068	1.690 (0.997, 2.866)	0.052
Typical rhythm	0.113 (−0.048, 0.274)	0.169	1.469 (0.893, 2.417)	0.130
Severely disrupted	0.587 (0.234, 0.939)	0.001	2.927 (1.224, 6.998)	0.016
RAR phenotype × CPDQS				
Robust rhythm + Higher CPDQS	Reference	—	Reference	—
Robust rhythm + Lower CPDQS	0.038 (−0.190, 0.266)	0.741	1.179 (0.560, 2.483)	0.665
Inter-daily instability + Higher CPDQS	−0.025 (−0.502, 0.451)	0.917	1.031 (0.243, 4.383)	0.967
Inter-daily instability + Lower CPDQS	0.103 (−0.287, 0.493)	0.604	1.680 (0.548, 5.145)	0.364
Fragmented low-amplitude + Higher CPDQS	0.157 (−0.084, 0.397)	0.202	1.544 (0.761, 3.133)	0.228
Fragmented low-amplitude + Lower CPDQS	0.255 (−0.033, 0.542)	0.082	2.294 (1.036, 5.079)	0.041
Typical rhythm + Higher CPDQS	0.014 (−0.196, 0.224)	0.897	1.136 (0.561, 2.302)	0.723
Typical rhythm + Lower CPDQS	0.257 (0.058, 0.455)	0.011	2.220 (1.192, 4.136)	0.012
Severely disrupted + Higher CPDQS	0.590 (0.106, 1.075)	0.017	3.850 (1.077, 13.763)	0.038
Severely disrupted + Lower CPDQS	0.631 (0.154, 1.109)	0.010	2.920 (0.911, 9.354)	0.071

Note: BCH, Bolck–Croon–Hagenaars; CI, confidence interval; CPDQS, China Prime Diet Quality Score; OR, odds ratio; RAR, rest–activity rhythm. Models were adjusted for age, sex, education, living arrangement, body mass index, smoking status, chronic disease burden, total energy intake, sleep duration, and sleep efficiency.

Table S8. Sensitivity analysis of the joint associations between diet quality and RAR phenotypes with a modified Fried frailty score excluding the low physical activity component.

Joint category	β (95% CI)	*P* value
Robust + Higher diet	Reference	—
Robust + Lower diet	0.079 (−0.090, 0.249)	0.358
Typical + Higher diet	−0.012 (−0.147, 0.123)	0.863
Typical + Lower diet	0.200 (0.051, 0.348)	0.008
Instability + Higher diet	−0.055 (−0.317, 0.208)	0.683
Instability + Lower diet	0.123 (−0.141, 0.387)	0.362
Fragmented + Higher diet	0.108 (−0.072, 0.288)	0.240
Fragmented + Lower diet	0.176 (−0.008, 0.361)	0.060
Severe + Higher diet	0.362 (0.012, 0.713)	0.043
Severe + Lower diet	0.472 (0.072, 0.873)	0.021

Table S9. Sex-stratified associations of joint diet quality and rest–activity rhythm phenotypes with frailty.

**Joint phenotype**	**Male (n = 525)**	**Female (n = 678)**
**β (95% CI) for linear regression**		
Robust rhythm + Higher diet quality	Reference	
Robust rhythm + Lower diet quality	0.014 (−0.279, 0.308)	0.154 (−0.048, 0.357)
Typical rhythm + Higher diet quality	−0.039 (−0.286, 0.208)	0.059 (−0.090, 0.208)
Typical rhythm + Lower diet quality	0.148 (−0.110, 0.405)	**0.296 (0.124, 0.468)**
Inter-daily instability + Higher diet quality	0.029 (−0.506, 0.564)	0.045 (−0.240, 0.330)
Inter-daily instability + Lower diet quality	−0.169 (−0.526, 0.189)	**0.509 (0.094, 0.924)**
Fragmented low-amplitude + Higher diet quality	−0.034 (−0.314, 0.247)	**0.255 (0.009, 0.502)**
Fragmented low-amplitude + Lower diet quality	0.120 (−0.204, 0.445)	**0.330 (0.083, 0.576)**
Severely disrupted + Higher diet quality	0.267 (−0.189, 0.722)	**1.003 (0.140, 1.866)**
Severely disrupted + Lower diet quality	0.516 (−0.139, 1.172)	**0.673 (0.088, 1.258)**
P for overall interaction	0.508	
**OR (95% CI) for ordered logistic regression**		
Robust rhythm + Higher diet quality	Reference	
Robust rhythm + Lower diet quality	1.130 (0.498, 2.565)	1.725 (0.802, 3.710)
Typical rhythm + Higher diet quality	0.916 (0.463, 1.811)	1.370 (0.721, 2.601)
Typical rhythm + Lower diet quality	1.496 (0.745, 3.004)	**2.872 (1.490, 5.535)**
Inter-daily instability + Higher diet quality	0.886 (0.253, 3.106)	1.589 (0.464, 5.444)
Inter-daily instability + Lower diet quality	0.657 (0.217, 1.995)	**5.712 (1.743, 18.720)**
Fragmented low-amplitude + Higher diet quality	0.937 (0.440, 1.997)	2.024 (0.922, 4.445)
Fragmented low-amplitude + Lower diet quality	1.355 (0.590, 3.113)	**3.266 (1.505, 7.089)**
Severely disrupted + Higher diet quality	1.384 (0.468, 4.096)	**23.178 (1.588, 338.303)**
Severely disrupted + Lower diet quality	1.678 (0.423, 6.654)	**4.820 (1.038, 22.379)**
P for overall interaction	0.448	

Note: Models were adjusted for continuous age, education, living arrangement, body mass index, smoking status, comorbidity, total energy intake, sleep duration, and sleep efficiency. Bold values indicate statistical significance at $P < 0.05$.

Table S10. Age-stratified associations of joint diet quality and rest–activity rhythm phenotypes with frailty.

**Joint phenotype**	**Age <75 (n = 839)**	**Age ≥75(n = 364)**
**β (95% CI) for linear regression**		
Robust rhythm + Higher diet quality	Reference	
Robust rhythm + Lower diet quality	0.096 (−0.089, 0.280)	−0.025 (−0.488, 0.439)
Typical rhythm + Higher diet quality	−0.018 (−0.165, 0.130)	0.092 (−0.273, 0.457)
Typical rhythm + Lower diet quality	**0.189 (0.024, 0.354)**	0.290 (−0.075, 0.656)
Inter-daily instability + Higher diet quality	0.021 (−0.364, 0.405)	−0.073 (−0.561, 0.416)
Inter-daily instability + Lower diet quality	0.210 (−0.124, 0.545)	−0.074 (−0.619, 0.471)
Fragmented low-amplitude + Higher diet quality	0.030 (−0.181, 0.241)	0.276 (−0.130, 0.682)
Fragmented low-amplitude + Lower diet quality	**0.280 (0.035, 0.525)**	0.196 (−0.210, 0.603)
Severely disrupted + Higher diet quality	0.472 (−0.033, 0.976)	0.622 (−0.135, 1.379)
Severely disrupted + Lower diet quality	0.531 (−0.112, 1.174)	**0.700 (0.045, 1.354)**
P for overall interaction	0.548	
**OR (95% CI) for ordered logistic regression**		
Robust rhythm + Higher diet quality	Reference	
Robust rhythm + Lower diet quality	1.405 (0.766, 2.579)	0.990 (0.271, 3.622)
Typical rhythm + Higher diet quality	0.991 (0.587, 1.672)	1.592 (0.567, 4.469)
Typical rhythm + Lower diet quality	**1.789 (1.049, 3.049)**	2.823 (0.988, 8.068)
Inter-daily instability + Higher diet quality	0.890 (0.275, 2.879)	1.704 (0.337, 8.616)
Inter-daily instability + Lower diet quality	2.341 (0.912, 6.006)	0.888 (0.158, 5.004)
Fragmented low-amplitude + Higher diet quality	1.130 (0.583, 2.193)	2.305 (0.777, 6.836)
Fragmented low-amplitude + Lower diet quality	**2.179 (1.107, 4.289)**	2.501 (0.808, 7.742)
Severely disrupted + Higher diet quality	3.480 (0.889, 13.620)	3.014 (0.523, 17.355)
Severely disrupted + Lower diet quality	2.048 (0.583, 7.187)	4.652 (0.764, 28.337)
P for overall interaction	0.444	

Note: Models were adjusted for continuous age, education, living arrangement, body mass index, smoking status, comorbidity, total energy intake, sleep duration, and sleep efficiency. Bold values indicate statistical significance at $P < 0.05$.

Table S11. Differences between in included and excluded participants due to missing data.

Characteristics		Total sample aged 65 to 85 (N=1887)	Included (n=1203)	Excluded due to missing data (n=684)	*P*-value
Age, years		72.58 (4.23)	72.49 (4.17)	72.74 (4.34)	0.313
Sex	Male	900 (47.7%)	525 (43.6%)	375 (54.8%)	<0.001
	Female	987 (52.3%)	678 (56.4%)	309 (45.2%)	
BMI, kg/m²		24.51 (3.59)	24.56 (3.58)	24.40 (3.61)	0.364
Education	Below primary school	1141 (61.4%)	728 (60.5%)	413 (63.0%)	0.083
	Primary school	328 (17.6%)	231 (19.2%)	97 (14.8%)	
	Middle school	293 (15.8%)	190 (15.8%)	103 (15.7%)	
	High school or higher	97 (5.2%)	54 (4.5%)	43 (6.6%)	
Living alone	No	1514 (81.4%)	970 (80.6%)	544 (82.9%)	0.313
	Yes	345 (18.6%)	233 (19.4%)	112 (17.1%)	
Smoking status	Current smoker	354 (19.1%)	211 (17.5%)	143 (22.1%)	0.002
	Former smoker	393 (21.2%)	236 (19.6%)	157 (24.3%)	
	Non-smoker	1103 (59.6%)	756 (62.8%)	347 (53.6%)	
Chronic disease status	No chronic condition	421 (22.7%)	283 (23.5%)	138 (21.1%)	0.363
	One chronic condition	579 (31.2%)	379 (31.5%)	200 (30.5%)	
	Two or more chronic conditions	858 (46.2%)	541 (45.0%)	317 (48.4%)	

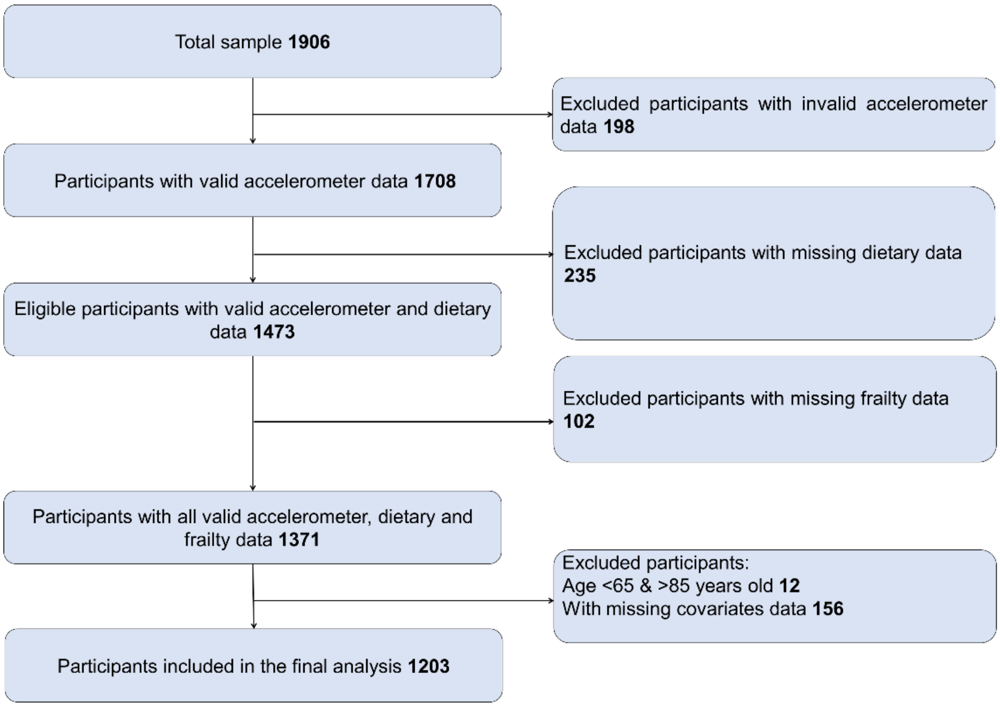

Figure S1. Participant flow diagram.
